# ASO Author Reflections: Benefit of Adjuvant Radiotherapy for Clinical Outcome in Patients with Soft Tissue Sarcoma

**DOI:** 10.1245/s10434-018-6818-6

**Published:** 2018-10-08

**Authors:** R. L. Haas, J. Szkandera

**Affiliations:** 1 Department of Radiotherapy, The Netherlands Cancer Institute-Antoni van Leeuwenhoek Hospital, Amsterdam, The Netherlands; 2 Department of Radiotherapy, The Leiden University Medical Center, Leiden, The Netherlands; 3 Division of Clinical Oncology, Department of Medicine, Medical University of Graz, Graz, Austria

## Past

Radiotherapy (XRT) improves local control and plays an essential part in the reduction of extensive surgical procedures in the management of patients with soft tissue sarcoma (STS). Historically, surgery is performed first followed, on indication, by adjuvant XRT (AXRT), balancing the gain in local control to toxicity. A landmark study by Rosenberg et al. demonstrated no statistically significant difference in local recurrence (LR), disease-free survival (DFS), and overall survival (OS) between STS patients after amputation and patients with limb-sparing surgery and AXRT.^1^ With respect to LR, these results have been confirmed in various studies. They have sparked a revolution, enabling less mutilating surgery and more limb conservation without compromising LR risk. However, it is not fully clarified whether AXRT also affects systemic outcomes, such as DM and OS, because conflicting results have been reported here. This study was designed to determine the impact of AXRT on LR, DM, and OS in STS patients after surgery using retrospective observational data.

## Present

A statistically significant reduction of the risk of LR of more than 50% by AXRT was demonstrated in this study.^2^ Furthermore, the study suggests a nonsignificant reduction in the risk of DM and subsequent death in favour of AXRT in STS patients.^2^ In a subgroup analysis that included only STS patients with high-grade (G3) tumors, a significant association between AXRT and improved OS was observed. However, this result might be due to residual confounding.^2^ These findings indicate that the application of AXRT after curative resection of STS significantly improves local control. However, for systemic control and survival, the role of AXRT is limited. The results of the study emphasize the value of XRT in the treatment of STS patients, which also is reflected by the current ESMO and NCCN guidelines that suggest to combine surgery and XRT for large and/or deep seated and/or intermediate to high-grade extremity STS.^3^,^4^

## Future

Prospective studies are needed to evaluate further the impact of XRT on clinical outcome in STS patients. Concerning neoadjuvant XRT, with the sarcoma mass still in situ, clinical trials should be designed with appropriate translational side studies in order to distinguish the more radiation sensitive subtypes. Obviously, STS represents many different tumours, each with their own clinical behaviour. Currently, all are irradiated to a uniform schedule.^5^ Combination regimens of XRT with modern targeted agents have been performed or are currently accruing patients. Sophisticated XRT techniques enable the delivery of the lowest radiation dose to normal sarcoma-surrounding tissues. For the future, taking individual patient selection criteria, trial availability, and anticipation of both acute toxicities and long-term morbidities into account, the patient shared decision making of experienced multidisciplinary sarcoma teams preferably moves towards an increased delivery of neoadjuvant XRT. From a radiotherapeutic setting, the main focus of ongoing research includes improved targeting of therapy to minimize toxicity and maximize disease control.
